# ML-GAP: machine learning-enhanced genomic analysis pipeline using autoencoders and data augmentation

**DOI:** 10.3389/fgene.2024.1442759

**Published:** 2024-09-25

**Authors:** Melih Agraz, Dincer Goksuluk, Peng Zhang, Bum-Rak Choi, Richard T. Clements, Gaurav Choudhary, George Em Karniadakis

**Affiliations:** ^1^ Division of Applied Mathematics, Brown University, Providence, RI, United States; ^2^ Department of Statistics, Giresun University, Giresun, Türkiye; ^3^ Department of Biostatistics, Erciyes University, Kayseri, Türkiye; ^4^ Vascular Research Laboratory, VA Providence Healthcare System, Providence, RI, United States; ^5^ Division of Cardiology, Department of Medicine, Alpert Medical School of Brown University, Providence, RI, United States; ^6^ Cardiovascular Research Center, Rhode Island Hospital, Providence, RI, United States; ^7^ Department of Biomedical and Pharmaceutical Sciences, University of Rhode Island College of Pharmacy, South Kingston, RI, United States; ^8^ School of Engineering, Brown University, Providence, RI, United States

**Keywords:** RNA-seq, differential expression, mixup, machine learning, feature selection

## Abstract

**Introduction:**

The advent of RNA sequencing (RNA-Seq) has significantly advanced our understanding of the transcriptomic landscape, revealing intricate gene expression patterns across biological states and conditions. However, the complexity and volume of RNA-Seq data pose challenges in identifying differentially expressed genes (DEGs), critical for understanding the molecular basis of diseases like cancer.

**Methods:**

We introduce a novel Machine Learning-Enhanced Genomic Data Analysis Pipeline (ML-GAP) that incorporates autoencoders and innovative data augmentation strategies, notably the MixUp method, to overcome these challenges. By creating synthetic training examples through a linear combination of input pairs and their labels, MixUp significantly enhances the model’s ability to generalize from the training data to unseen examples.

**Results:**

Our results demonstrate the ML-GAP’s superiority in accuracy, efficiency, and insights, particularly crediting the MixUp method for its substantial contribution to the pipeline’s effectiveness, advancing greatly genomic data analysis and setting a new standard in the field.

**Discussion:**

This, in turn, suggests that ML-GAP has the potential to perform more accurate detection of DEGs but also offers new avenues for therapeutic intervention and research. By integrating explainable artificial intelligence (XAI) techniques, ML-GAP ensures a transparent and interpretable analysis, highlighting the significance of identified genetic markers.

## 1 Introduction

RNA sequencing (RNA-Seq) employs advanced sequencing technology to identify the nucleotide sequence of RNA molecules and measure the abundance of specific RNA types within RNA molecule populations (Deshpande et al., 2023). Typically, the main goal of RNA-Seq analysis is to detect genes that are expressed differently under various biological circumstances (Piao and Ryu, 2017). Traditional RNA-Seq analysis methodologies, while effective, often face limitations in identifying differentially expressed genes (DEGs) that are crucial for understanding the molecular underpinnings of diseases, including cancer. This challenge is magnified by the complexity and sheer volume of data generated, necessitating advanced analytical approaches to fully harness the potential of RNA-Seq data.

Recent research has established that the application of machine learning (ML) algorithms to RNA-Seq data can effectively classify different problems based on gene expression profiles. Dag et al. (Dag et al., 2023) introduced GeneSelectML, an open-source web-based tool for gene selection from RNA-Seq data using ML algorithms. It features 6 ML algorithms, pre-processing steps, and graphical outputs like heatmaps and network plots, alongside gene ontology analysis for DEGs. Demonstrated on Alzheimer’s RNA-seq data, GeneSelectML aids in identifying potential biomarkers by employing multiple ML algorithms simultaneously for gene selection. This tool combines pre-processing, visualization and ML methods. Stathopoulou et al. (Stathopoulou et al., 2024) investigated the overlap of ML algorithms in RNA-seq analysis for estimating gene expression across various cancer types. Using Random Forest and Gradient Boosting algorithms, their research demonstrated the reproducibility and overlap in identifying significant DEGs, enhancing the understanding of genes’ roles in cancer development. Their findings highlight the efficacy of combining ML with RNA-seq to improve the identification of critical DEGs, contributing valuable insights into cancer biology and potentially improving diagnostic and therapeutic strategies. Wang et al. (Wang et al., 2018) identified important features with three feature selection algorithms (Information Gain, Correlation Feature Selection, and ReliefF). They employed five widely used classifiers (Logistic Regression, Classification via Regression, Random Forest, Logistic Model Trees, Random Subspace) to predict DEGs. Their study demonstrates the applicability of ML to improve the prediction and understanding of gene expression, specifically in relation to transcriptional regulation in response to ethylene in plant seedlings. Piao and Ryu (Piao and Ryu, 2017) introduced a novel approach for identifying DEGs in RNA-seq data by applying a feature selection method that utilizes symmetrical uncertainty for gene ranking and a predefined relevance threshold for selection. In their evaluation, the method achieved high sensitivity (0.986) and specificity (0.982) on large-sample datasets, outperforming traditional statistical approaches like edgeR (Robinson et al., 2009), DESeq (Love et al., 2014), and baySeq (Hardcastle and Kelly, 2010) in terms of Area Under the Curve values, with notable improvements observed even in challenging small-sample conditions. Arowolo et al. (Arowolo et al., 2020) developed a principal component analysis (PCA) model to enhance RNA-seq malaria vector data classification using k-nearest neighbour (KNN) and Decision Tree algorithms. The team aimed to address the challenge of high dimensionality in malaria vector RNA-seq data by employing PCA for feature extraction. This method effectively reduced the dataset’s complexity, making it more manageable for classification tasks. The classification performance was evaluated on a mosquito *Anopheles gambiae* RNA-Seq dataset, yielding accuracy rates of 86.7% for KNN and 83.3% for Decision Tree classifiers.

While the application of ML to RNA-Seq data shows promise, there are concerns regarding the interpretability of the models. According to Rudin (Rudin, 2019), black box ML models may not be suitable for high-stakes decisions in the context of medical research. Instead, Rudin suggests the use of interpretable models to ensure transparency and understanding of the underlying mechanisms (Rudin, 2019). This insight raises important considerations for the development and application of ML models in the context of RNA-Seq data analysis. The present study introduces a novel Machine Learning-Enhanced Genomic Analysis Pipeline (ML-GAP) utilizing autoencoders and data augmentation, aimed at addressing these challenges. By integrating ML techniques with autoencoders and data augmentation strategies, ML-GAP provides a new approach to enhance the detection of DEGs, particularly those elusive to conventional methods. Our pipeline not only facilitates a more in-depth analysis of RNA-Seq data but also aids in the identification of potential genes associated with clinical outcomes, thereby offering new avenues for medical research. The incorporation of explainable artificial intelligence (XAI) techniques distinguishes ML-GAP, enabling a transparent and interpretable analysis that underscores the significance of identified genetic markers. ML-GAP stands as a testament to the power of ML in genomic data analysis, setting a new standard for accuracy, efficiency, and insights in the field.

The paper is organized as follows. In Section 2, the materials and methods used in the study are presented, covering the comprehensive workflow of ML-GAP for RNA-Seq data. In Section 3, we present the findings from applying the ML-GAP to RNA-Seq data, highlighting the performance of our approach in identifying DEGs and showcasing the effectiveness of the MixUp method in improving model generalization. Additionally, the full forms of the abbreviations are listed in Supplementary Table S1.

## 2 Material and methods

### 2.1 Overview of the study

This study introduces ML-GAP, an approach specifically designed to address the challenges inherent in RNA-Seq data analysis. It leverages advanced ML techniques, including autoencoders and data augmentation strategies such as MixUp, to enhance the detection of DEGs. These methods not only improve the accuracy of gene expression analysis but also offer robustness against the noise and variability typical of RNA-Seq data. The pipeline is further refined by the incorporation of XAI techniques, ensuring that the analytical process remains transparent and the results interpretable. We constructed this pipeline using Python as the main programming language, integrating several libraries such as Scikit-learn for machine learning, SHAP and LIME for explainability, and other tools for genomic data preprocessing and analysis.

1. Count Data The initial step involves compiling a data matrix, denoted as X , which has dimensions p -by- n , representing genes -by- samples .
2. Data Preprocessing
   a. *Filtering:* Applying both low count filtering and zero-variance filter to refine the count data.
   b.*Normalization*: Employing DESeq median normalization to adjust the data.
   c.*Transformation*: Implementing variance stabilizing transformation to make the data more suitable for analysis.
3. Dimension Reduction
   a. Principal Component Analysis (PCA): PCA is employed to reduce the gene count in the genetic or molecular profiles dataset to 2000. This step significantly simplifies the complexity of the data, making it more manageable for subsequent analysis and improving interpretability.
   b.Dimension Reduction for Differentially Expressed Genes (DEGs): Following PCA, the gene count is further reduced to 200 features using differential expression analysis. This focused reduction selects genes that show statistically significant differences in expression associated with clinical outcomes, thus enhancing the potential for meaningful biological insights and therapeutic targets.
4. Machine Learning Performance After applying the dimensionality reduction techniques, we evaluated the performance of machine learning models using three distinct approaches: PCA and DEGs, Autoencoders, and Augmentation with MixUp. For each approach, we calculated several performance metrics, including Accuracy, Positive Predictive Value (PPV), Negative Predictive Value (NPV), Sensitivity, Specificity, and F1 Score. These metrics provide a comprehensive evaluation of the models’ effectiveness in identifying DEGs across our datasets. We optimized the model parameters using a 5-fold cross-validation grid search. To evaluate the model performance, we used an independent test set, splitting the dataset into training and testing sets with an 80/20 ratio using the train_test_split function from Scikit-learn.
   a. PCA and DEGs Approach: Dimensionality was first reduced via PCA and DEGs selection. Machine learning models were then applied to this refined dataset, and the performance was assessed based on the aforementioned metrics.
   b.Autoencoders Approach: Following dimensionality reduction via autoencoders, models were trained and evaluated similarly using the same set of metrics.
   c.Augmentation Approach (MixUp): The dataset was augmented using the MixUp technique before applying the machine learning models, which were evaluated using the same metrics to assess the impact of augmentation on model performance.
5. Explainable Machine Learning (XAI)
   a. *SHAP (SHapley Additive exPlanations)* SHAP is employed to determine the influence of each gene on the model’s output, thereby enhancing the interpretability of the model’s decisions. It identifies the top-10 features based on the performance evaluation.
   b.*LIME (Local Interpretable Model-agnostic Explanations)* LIME elucidates model predictions by approximating the model locally with an interpretable surrogate, which helps in understanding the impact of various features on the model’s decisions. Top-10 features are identified following the analysis of model performance.
   c. *Variable Importance (VarImp)* This technique highlights the genes that significantly influence predictions, underlining their biological importance. VarImp is used to determine top-10 features after applying ML models and evaluating their performance.
6. Biological Validation
 a. *Graphical Representations* Visual representations are created such as Volcano plots and Ven diagrams.
 b.*Gene Ontology* The functionality of selected genes is compared with existing literature to validate their roles and relevance in biological processes and disease mechanisms.

### 2.2 Dataset and preprocesisng

We conducted an analysis utilizing two RNA-Seq datasets pertaining to Renal Cell Carcinoma (RCC), sourced from The Cancer Genome Atlas (TCGA) network.

#### 2.2.1 Renal cell carcinoma (RCC)

From TCGA, we obtained sequencing reads for 20,531 recognized human RNAs from a cohort of 1,020 patients diagnosed with RCC. These patients were classified into three predominant subtypes: kidney renal papillary cell carcinoma (KIRP), kidney renal clear cell carcinoma (KIRC), and kidney chromophobe carcinoma (KICH), with respective sample counts of 606, 323, and 91. In the RCC dataset, we excluded data labeled as KICH, maintaining only the records for KIRC and KIRP patients, culminating in a final tally of 929 observations.

#### 2.2.2 Lung cancer data

We utilized data on lung cancer obtained from the TCGA platform. This dataset includes the mapped read counts for 20,531 recognized human RNAs from 1,128 patients with lung cancer. These patients were classified into two specific lung cancer types: lung adenocarcinoma (LUAD) and lung squamous cell carcinoma (LUSC), with respective sample sizes of 576 and 552.

**FIGURE 1:**
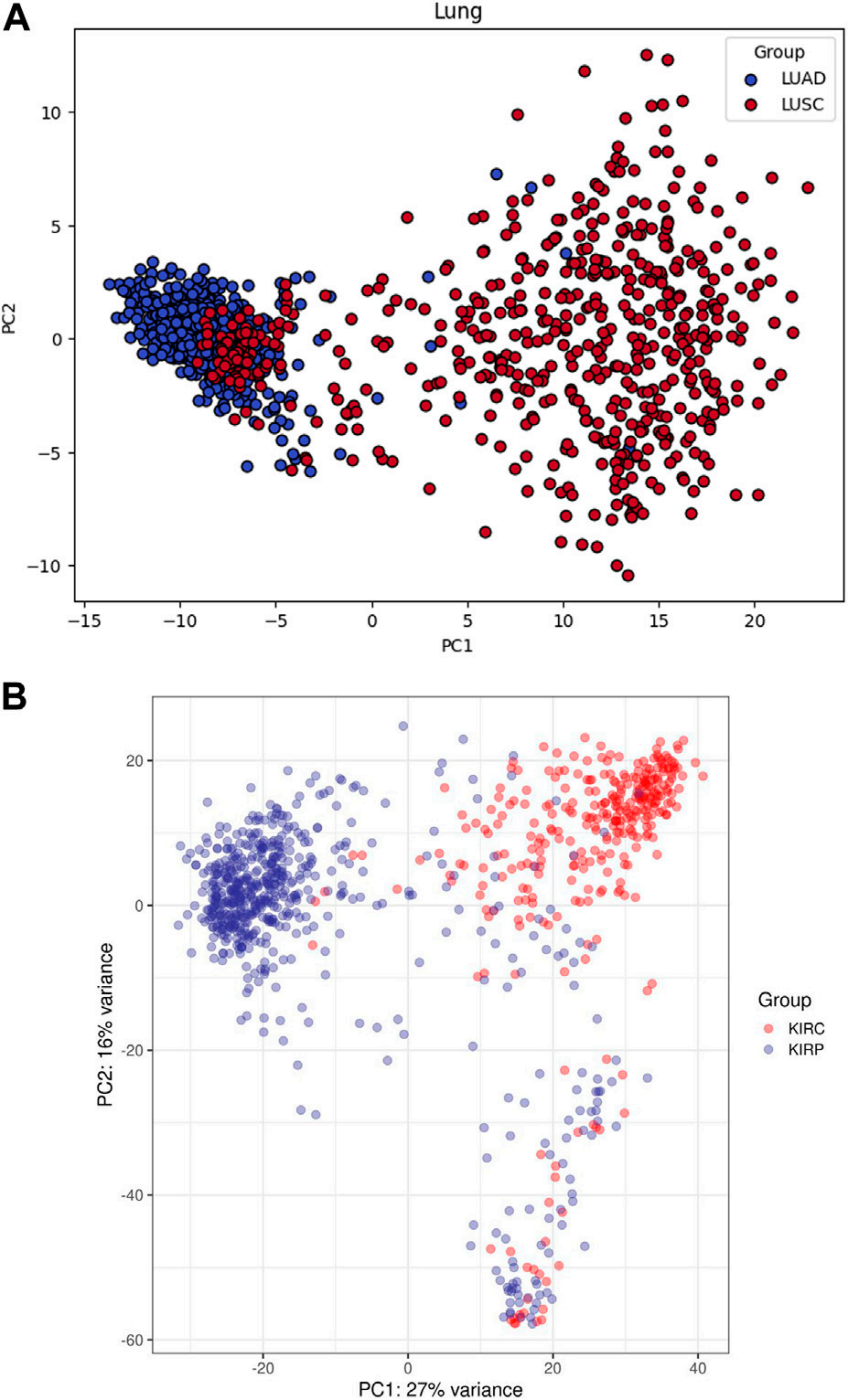
Principal components plots for **(A)** Lung and **(B)** RCC data.

Principal components plots for lung and RCC data, shown in Figure 1, illustrate the separation of samples based on the first two principal components. These plots highlight the inherent variance within the datasets and the effectiveness of the preprocessing steps. The dimensionality reduction achieved through PCA is crucial for subsequent analyses, as it highlights the distinct clustering of the different cancer subtypes, thereby confirming the integrity and quality of our dataset.

##### 2.2.3 Data preprocessing

**FIGURE 2:**
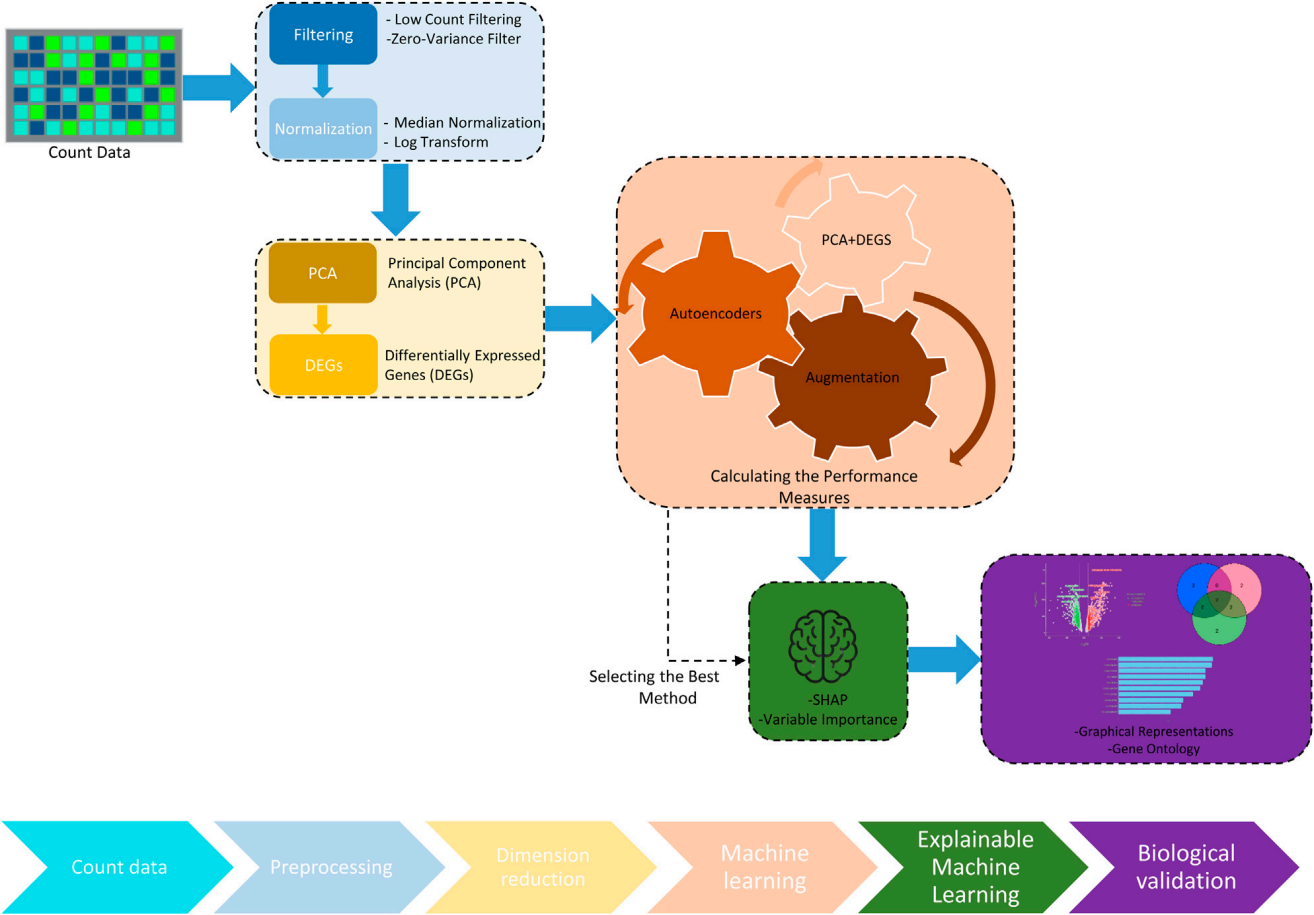
Workflow of the data processing and the pipeline in the proposed methodology. The workflow utilized for processing and analyzing transcriptomics data. Starting with raw count data, the process involves preprocessing steps such as filtering and normalization, followed by dimension reduction through principal component analysis (PCA). The analysis identifies differentially expressed genes (DEGs), which are then processed through ML techniques, including PCA and DEGs and Autoencoders, to measure and enhance algorithm performance. The pipeline integrates data augmentation techniques to improve model robustness and employs explainable artificial intelligence (XAI) tools like SHAP to interpret the model outcomes. The workflow concludes with molecular biology validation to confirm the biological relevance of the findings.

RNA-Seq data inherently presents an abundance of zero values and encompasses variations between samples stemming from experimental procedures. The preprocessing steps outlined in Figure 2 are employed to mitigate the impact of low-quality features and inter-sample variations. Initially, features with exceedingly low counts were excluded through near-zero variance filtering (Kuhn, 2008). Subsequently, a normalization process was executed on the pre-filtered raw counts before progressing to downstream analyses. This step aimed to mitigate the influence of sequencing depth, technical variation, and potential biases, while retaining the inherent biological variations among samples. To achieve this, we implemented the median-ratio normalization method from the DESeq2 package (Love et al., 2014), chosen for its resilience against outliers and its efficacy in eliminating technical variations across samples. We calculated the size factor $s_{j}$ using DESeq normalization, as expressed by the following Equation 1:

$$s_{j}=\frac{m_{j}}{\sum_{j=1}^{n}m_{j}},m_{j}=\text{median}{\left\{\frac{x_{ij}}{G_{i}}\right\}}_{i:G_{i}\neq 0},G_{i}={\left(\overset{n}{\underset{j=1}{\prod }}x_{ij}\right)}^{1/n}$$ (1)

where $G_{i}$ represents the geometric mean of raw counts for the i -th feature. The quantity $m_{j}$ is computed over features having non-zero geometric means. Various alternative normalization techniques, such as the trimmed mean of M-values, upper quantile, Reads Per Kilobase per Million mapped reads (RPKM), and the logarithm of counts per million reads (log-CPM), are discussed in pertinent literature (Bullard et al., 2010; Mortazavi et al., 2008; Robinson and Oshlack, 2010). This study employed the ‘variance stabilizing transformation (VST)’ (Anders and Huber, 2010; Love et al., 2014), as implemented in the DESeq2 package. The primary objective was to mitigate the dependence between mean and variance in normalized counts, thereby approximating the data to normality and rendering it less skewed. It is imperative to acknowledge that following the VST transformation, the variances exhibit approximate independence from the mean. Nevertheless, it is crucial to recognize that these variances remain unequal across all genes, potentially leading to the presence of outliers within the dataset (Zwiener et al., 2014).

In the preprocessing step, we transfer the data from raw counts to normalized counts. Given that RNA-Seq data contains many features and few observations (n≪p) , it becomes imperative to meticulously select a subset of features that exhibit associations with the outcome and actively contribute to the efficacy of the fitted model. Consequently, we are sequentially applying PCA and differential expression analysis.

### 2.3 Dimension reduction

#### 2.3.1 Principal component analysis (PCA)

Principal Component Analysis (PCA) is a statistical technique used in data analysis and ML to emphasize variation and extract strong patterns, with its modern version formalized by Hotelling (Hotelling, 1933). It simplifies complex genetic information by transforming it into a lower-dimensional space, revealing key variables (principal components). By highlighting significant relationships in high-dimensional data, PCA aids in visualizing and analyzing genetic patterns and biological processes. This study employs PCA after normalizing RNA-Seq data to reduce its dimensionality.

#### 2.3.2 Differentially Expressed Genes (DEGs)

The identification of differentially expressed genes (DEGs) is crucial in bioinformatics to detect genes with statistically significant differences in expression levels across various sample groups. After applying PCA for dimensionality reduction, further analysis focuses on identifying DEGs to gain insights into genetic expression changes linked to clinical outcomes. This dual approach ensures that features selected for ML models are highly informative, both statistically and biologically, thereby reducing complexity while highlighting potential therapeutic targets.

### 2.4 Machine learning model performance

#### 2.4.1 Autoencoders

**FIGURE 3:**
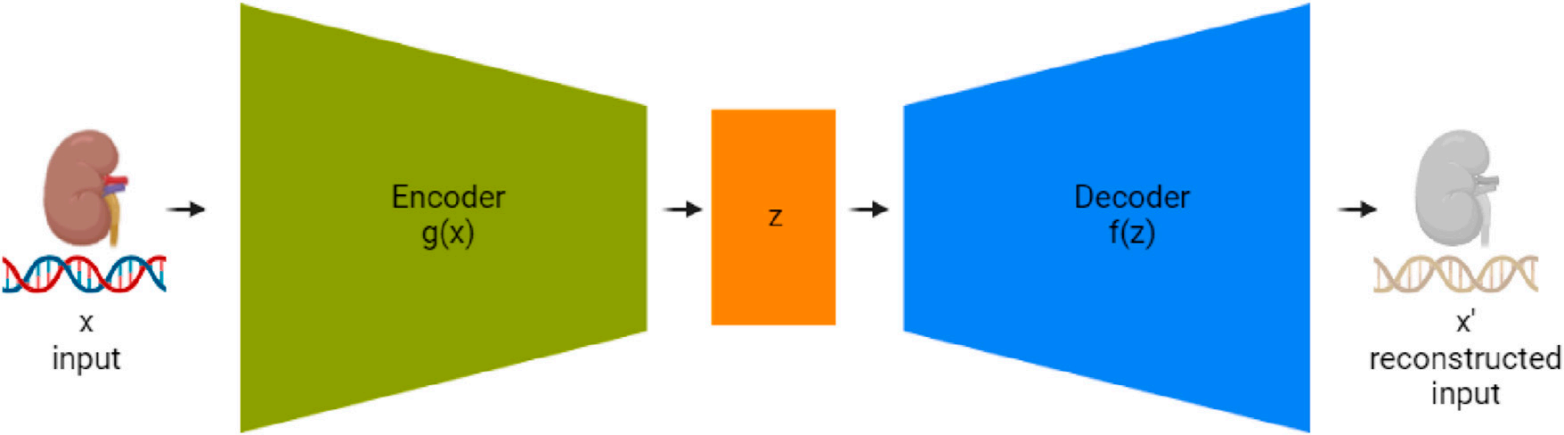
Detailed schematic of an autoencoder network for RNA sequencing data compression and reconstruction. The ‘Encoder’ stage (g(x)) processes the input high-dimensional RNAseq data, compressing it into a condensed representation at the ‘Bottleneck Layer’ (z). Subsequently, the ‘Decoder’ stage (f(z)) aims to reconstruct the RNAseq data from this lower-dimensional representation to an output (x’) that is as similar to the original input as possible. This process enables the reduction of data complexity and noise, facilitating more efficient storage and analysis.

Autoencoders, first introduced by McClelland et al. (1987), are a type of neural network used in unsupervised learning, designed to recreate their input. Their primary function is to learn useful features or representations within the data. An autoencoder typically consists of three key components: an encoder, a compressed representation (also known as the bottleneck or latent layer), and a decoder as seen in Figure 3. The encoder condenses the data into a reduced-dimensional space, and the decoder restores the data to its original shape. In our study, we employed an autoencoder neural network structure to explore dimensionality reduction within our dataset, which was split into training and test sets. The input layer of the autoencoder consisted of 200 neurons, corresponding to the dimensionality of the data in the training set. This was followed by an encoder layer with 128 neurons and a hidden layer with 72 neurons, representing the compressed representation of the input data. The decoder part of the network then aimed to reconstruct the input data from this reduced 72-neuron representation. By training the autoencoder on the training set and evaluating its performance on the test set, we were able to assess the model’s ability to reduce the dimensionality of the data from 200 to 72 while still retaining the essential characteristics necessary for reconstruction. The observations in the test set that resulted in the lowest reconstruction error, particularly the 72 with the most minimal error, were identified as those best represented by the autoencoder, demonstrating the model’s effectiveness in dimensionality reduction.

#### 2.4.2 Augmentation (MixUp)

Zhang et al. (Zhang et al., 2017) introduced an advanced data augmentation technique, called MixUp, devised to address the issue of class imbalance by generating new samples through a strategic combination of data points. This technique is grounded on the Equation 2:

$$\tilde{x}=\lambda x_{i}+\left(1-\lambda \right)x_{j},\;\tilde{y}=\lambda y_{i}+\left(1-\lambda \right)y_{j},$$ (2)

where $\tilde{x}$ and $\tilde{y}$ denote the synthesized input and output pairs, respectively. The coefficient λ is sampled from the Beta distribution, Beta(α,α) , highlighting the significance of α as a crucial hyperparameter in modulating the mixing intensity. The inputs $x_{i}$ and $x_{j}$ , along with their corresponding outputs $y_{i}$ and $y_{j}$ , are selected from distinct samples within the dataset. A distinctive aspect of our implementation of the MixUp algorithm is its application exclusively to the minority class, aimed at improving the representation of underrepresented categories within the dataset without altering the distribution of the majority class. This targeted approach towards the minority class is designed to enhance the model’s sensitivity to these categories, potentially leading to improved fairness and accuracy in the model’s predictions across varied datasets.

#### 2.4.3 Machine learning model performance

In the analysis of RCC and lung cancer data, both Random Forest and XGBoost ML algorithms were utilized to develop predictive models. To optimize the performance of these models, a comprehensive grid search was conducted to fine-tune their hyperparameters. This involved the use of repeated k-fold cross-validation, specifically partitioning the data into five folds, to ensure a robust evaluation of the model’s performance across various subsets of the data. The grid search method, executed with a 5-fold cross-validation approach, is crucial for verifying the generalizability and efficacy of the ML models on unseen data, thereby ensuring that the selected models are well-suited for predicting outcomes in RCC and lung cancer cases based on their respective optimized parameters. We explain different algorithm steps in the following workflow as represented in Figure 2.

(a) *PCA and DEGs*: Initially, the dimensionality of the dataset was reduced using PCA and the identification of DEGs. This step was crucial for simplifying the dataset by focusing on the most informative features.
(b) *Autoencoders Approach*: Following the initial dimensionality reduction, autoencoders were utilized as an advanced technique to further compress the dataset into a more manageable size. This was particularly beneficial for capturing complex, nonlinear relationships within the data. ML models were then applied to this refined dataset, and their performance metrics were evaluated.
(c) *Augmentation Approach*: To augment the dataset post-initial dimensionality reduction (PCA and DEGs), an augmentation technique, specifically MixUp, was implemented. This approach not only reduced dimensionality but also increased the number of samples by blending observations. Subsequently, ML models were applied to this enhanced dataset, and their efficacy was assessed through various performance metrics.

### 2.5 Explainable Machine Learning (XAI)

Upon determining the most effective dimensionality reduction method—be it PCA and DEGs, Autoencoders, or Augmentation approaches, based on accuracy metrics—we proceeded to apply XAI techniques to elucidate the predictive models further. Specifically, we utilized SHAP, LIME, and VarImp analyses and selected the top-10 important features. These techniques were instrumental in identifying the most significant features contributing to the models’ predictions, thereby enhancing the transparency and interpretability of our ML models. This step is critical for understanding the underlying mechanisms of the models’ decisions, particularly in the complex domain of RCC and lung cancer prediction, where interpretability is as crucial as accuracy.

### 2.6 Biological Validation

#### 2.6.1 Graphical representations

Following the identification of effective features through the application of XAI techniques, we visually represented these selected features using Volcano plots and Venn diagrams. Volcano plots were employed to depict the statistical significance *versus* the magnitude of change of the features, offering a clear visualization of the most influential features identified by our models. Similarly, Venn diagrams were utilized to illustrate the overlap and uniqueness of significant features across different models or dimensionality reduction techniques.

#### 2.6.2 Gene ontology (GO)

In our analysis pipeline, we incorporated a Gene Ontology (GO) step to ascertain whether the selected features are relevant to lung cancer or RCC. This bioinformatics approach enables us to categorize the identified genes based on the related biological processes, cellular components, and molecular functions. By mapping the significant features to GO terms, we were able to validate the biological significance of these features in the context of lung cancer and RCC. This step is crucial for ensuring that the ML models are not only statistically robust but also biologically relevant, thereby enhancing the credibility and applicability of our findings in clinical settings. In this step, we checked the selected features that are related to the literature on lung cancer and listed the results listed in Table 2.

## 3 Results and discussion

In our study, as delineated in Figure 2, we initiated our data preprocessing by applying a “Filtering” step to the lung cancer and RCC count data. This filtering aimed at excluding features with low variability, specifically targeting those with negligible variance—often referred to as ‘near-zero variance’. This preliminary refinement was critical to ensure the integrity of our dataset, thereby preventing potential biases in the analysis due to superfluous or non-informative data. Subsequent to the filtering, we proceeded with the normalization of the data in the “Normalization” step. For this purpose, we employed a median-ratio normalization method, which is a part of the *DESeq2* bioinformatics tool. This particular normalization technique is known for its effectiveness in reducing technical biases without distorting the genuine biological differences between the samples. By employing this approach, we were able to achieve a normalized dataset that reliably reflects the underlying biological conditions. In the subsequent phase of our analysis, following the filtering and normalization steps, we employed “PCA” step as a dimensionality reduction technique. PCA is a robust statistical method that converts the data into a series of linearly independent variables called principal components. This technique enables us to reduce the complexity of our data by transitioning from a high-dimensional space—comprising approximately 19,000 variables—to a more manageable, lower-dimensional space. By doing so, PCA accentuates the most significant patterns and relationships within the data, which are essential for our further analysis. The dimension reduction accomplished through PCA is instrumental in enhancing computational efficiency and improving the interpretability of the dataset, thus preparing the ground for the subsequent identification of DEGs. Subsequent to dimensionality reduction through PCA, where we effectively condensed our dataset’s features from around 2000 to 200 features, we embarked on the critical phase of identifying DEGs in “DEGs” step. DEGs are genes that show statistically significant differences in expression levels between different biological states or experimental conditions. In our analysis, the reduced dimensionality facilitated a more targeted and efficient investigation into gene expression changes. By applying statistical tests to the principal components, we were able to detect genes whose expression levels were consistently altered across our sample groups. This identification of DEGs is a pivotal step in our research, as it lays the groundwork for understanding the molecular mechanisms that may underlie the biological phenomena under study. The DEGs serve as valuable markers for potential pathways that are activated or suppressed in response to specific conditions, providing insights that are essential for further biological interpretation and validation. After the filtering and dimension reduction steps, we compare the proposed methods with classic PCA and DEGs dimension reduction techniques in the Machine Learning Solution step. In this step, we compare three different methods as seen below.

1. Machine learning algorithms, including XGBoost and Random Forest, were applied to a dataset of 200 genes that had been refined through PCA and DEGs identification methods. The effectiveness of these algorithms was evaluated using various performance metrics.
2. Autoencoders were then employed to further reduce the dimensionality of the dataset, which resulted from PCA and DEGs methods, down to 72 features. Subsequently, the XGBoost and Random Forest algorithms were applied to this reduced dataset, and performance metrics were calculated to assess the impact of this dimensionality reduction.
3. The dataset of 200 genes, derived from PCA and DEGs methods, was augmented using the MixUp technique, which artificially expands the data pool without additional dimensionality reduction. This augmentation is intended to enhance the robustness and generalizability of the ML models. After augmentation, the same ML algorithms were re-applied, and the resulting performance metrics were examined to determine the effectiveness of this augmentation.

As detailed in Table 1, the MixUp technique, when applied to the RCC dataset, emerged as the most effective strategy across various metrics, particularly when used with the XGBoost model. This method achieved outstanding performance, with perfect scores for NPV (1.0000) and Recall (Sensitivity) (1.0000), indicating its accuracy in correctly identifying both true negatives and true positives. Additionally, MixUp delivered impressive results in terms of Accuracy (0.9946), Precision (PPV: 0.9915), and the F1 Score (0.9957), which highlights the balance between Precision and Recall.

**TABLE 1:** Comparative analysis of model evaluation metrics for XGBoost and Random Forest across different datasets: RCC and lung datasets. The performance metrics are shown for different data processing techniques including PCA & DEGs, Autoencoders, and MixUp augmentation.

XGBoost							
	Accuracy	Precision	NPV	Recall	Specificity	F1 Score	Time (s)
PCA and DEGs (Lung)	0.9690	0.9758	0.9608	0.9680	0.9703	0.9719	416.07
(1128×201)							
Autoencoders (Lung)	0.9601	0.9531	0.9694	0.9760	0.9406	0.9644	213.42
(1128×63)							
MixUp (Lung)	0.9823	0.9754	0.9904	0.9917	0.9717	0.9835	573.21
(2256×201)							
PCA and DEGs (RCC)	0.9624	0.9756	0.9365	0.9677	0.9516	0.9717	341.86
(929×201)							
Autoencoders (RCC)	0.9516	0.9528	0.9492	0.9758	0.9032	0.9641	173.84
(929×63)							
MixUp (RCC)	0.9946	0.9915	1.0000	1.0000	0.9857	0.9957	466.98
(1858 × s 201)							

Random Forest							
	Accuracy	Precision	NPV	Recall	Specificity	F1 Score	Time (s)
PCA and DEGs (Lung)	0.9602	0.9394	0.9894	0.992	0.9208	0.9650	421.96
(1128×201)							
Autoencoders (Lung)	0.9602	0.9462	0.9792	0.9840	0.9307	0.9647	348.23
(1128×63)							
MixUp (Lung)	0.9757	0.9636	0.9902	0.9917	0.9575	0.9774	742.91
(2256×201)							
PCA and DEGs (RCC)	0.9355	0.9444	0.9167	0.9597	0.8871	0.9520	749.18
(929×63)							
Autoencoders (RCC)	0.9570	0.9677	0.9355	0.9677	0.9355	0.9677	442.19
(929×63)							
MixUp (RCC)	0.9946	1.0000	0.9860	0.9913	1.0000	0.9957	766.60
(1858×201)							

Although the computational time for MixUp was higher due to the increased dataset size from synthetic data generation (as reflected in Table 1), the significant improvements in predictive performance more than justify this added cost. Particularly in cases where achieving a balance between Precision and Recall is critical, MixUp proved to enhance model robustness and overall classification performance. The technique’s superior results in handling imbalanced data, especially in the RCC dataset, underscore its potential to improve model reliability across various performance metrics.

**FIGURE 4:**
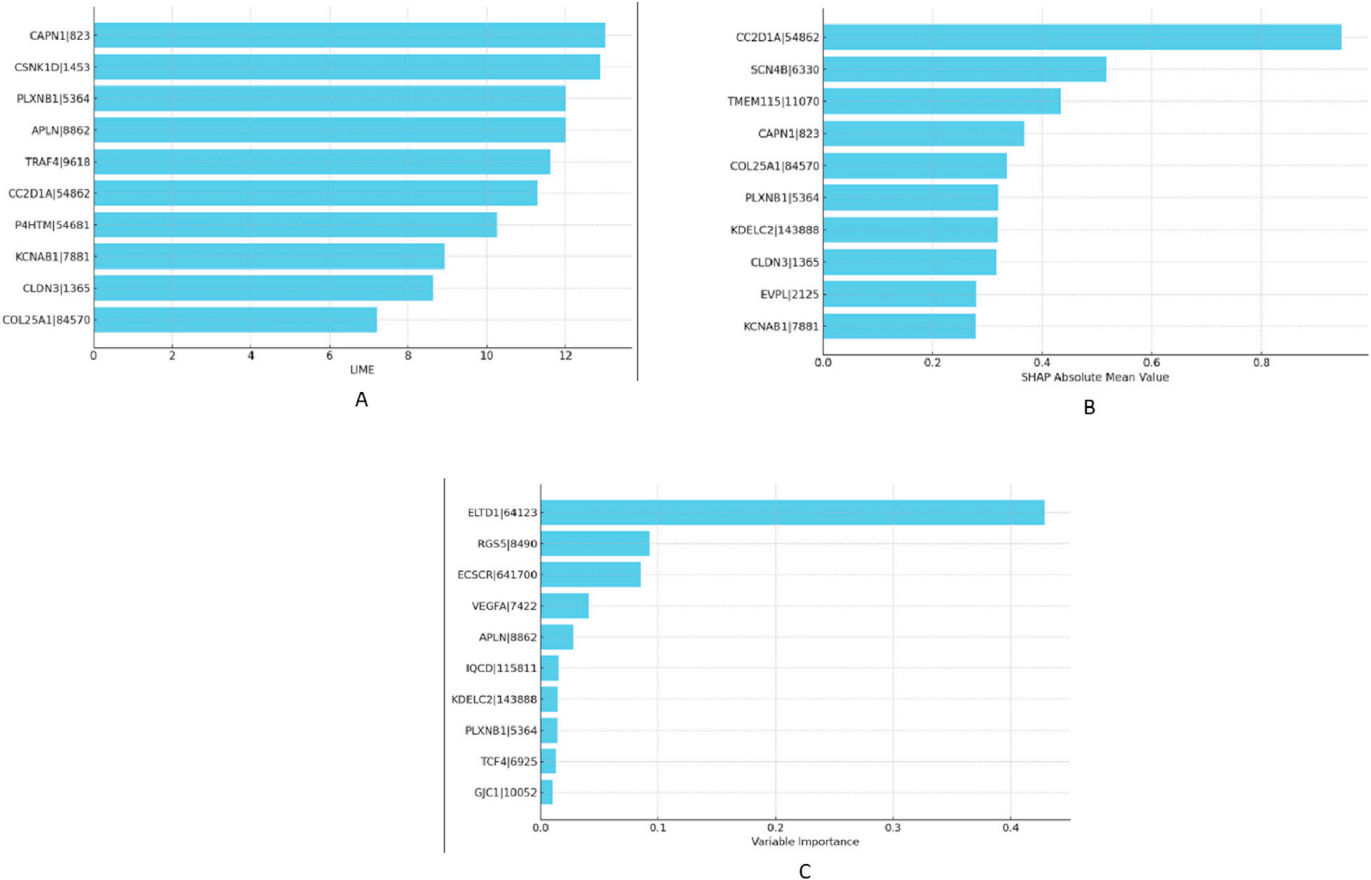
Comparative visualization of feature importances as determined by XGBoost for RCC dataset: **(A)** LIME scores, **(B)** SHAP values, and **(C)** variable importance metrics.

Given the superior results yielded by the MixUp method, we proceeded to identify the most impactful features affecting our RNA-Seq data within this framework in “XAI” step. To accomplish this, we calculated VarImp using the Random Forest and XGBoost algorithms applied to both lung and RCC datasets. We further enriched our analysis by implementing SHAP and LIME methodologies to ascertain the top-10 most essential genes in our model’s performance. The selected genes were not only prominent based on variable importance rankings but also stood out in SHAP and LIME analyses, confirming their significant role in the predictive model. We have listed the top-10 features obtained from the XGBoost and Random Forest models—namely SHAP, LIME, and VarImp—in Figure 4.

**FIGURE 5:**
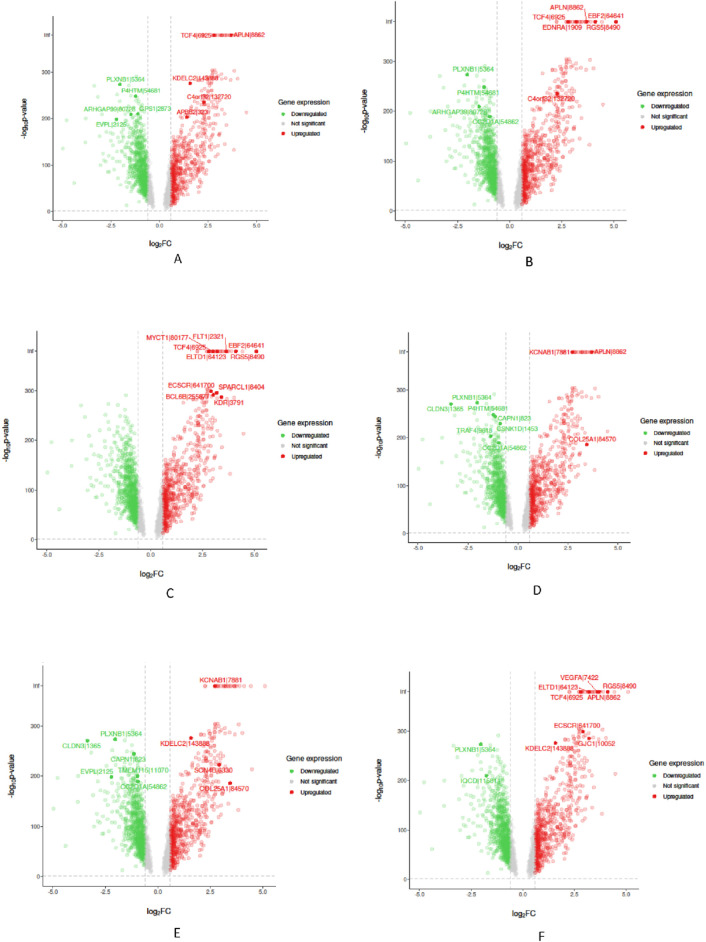
Volcano plots for Random Forest (RF) and eXtreme Gradient Boosting (XGB) models displaying selected feature importance in RCC data. Each plot utilizes a different interpretability method, namely LIME, SHAP, and Variable Importance (VarImp), to highlight the most influential features. The *x*-axis shows the log fold change (logFC), indicating the magnitude of change in gene expression, while the *y*-axis represents the negative logarithm of the p-value (−log10 p-value), denoting statistical significance. The plots differentiate between significant (red), non-significant (green), and unregulated (gray) gene expressions. **(A)** RF LIME - Random Forest model feature importance using LIME explanation method. **(B)** RF SHAP - Random Forest model feature importance using SHAP values for explanation. **(C)** RF VarImp - Random Forest model feature importance using Variable Importance. **(D)** XGB LIME - eXtreme Gradient Boosting model feature importance using LIME explanation. **(E)** XGB SHAP - eXtreme Gradient Boosting model feature importance using SHAP values for explanation. **(F)** XGB VarImp - eXtreme Gradient Boosting model feature importance using Variable Importance.

**FIGURE 6:**
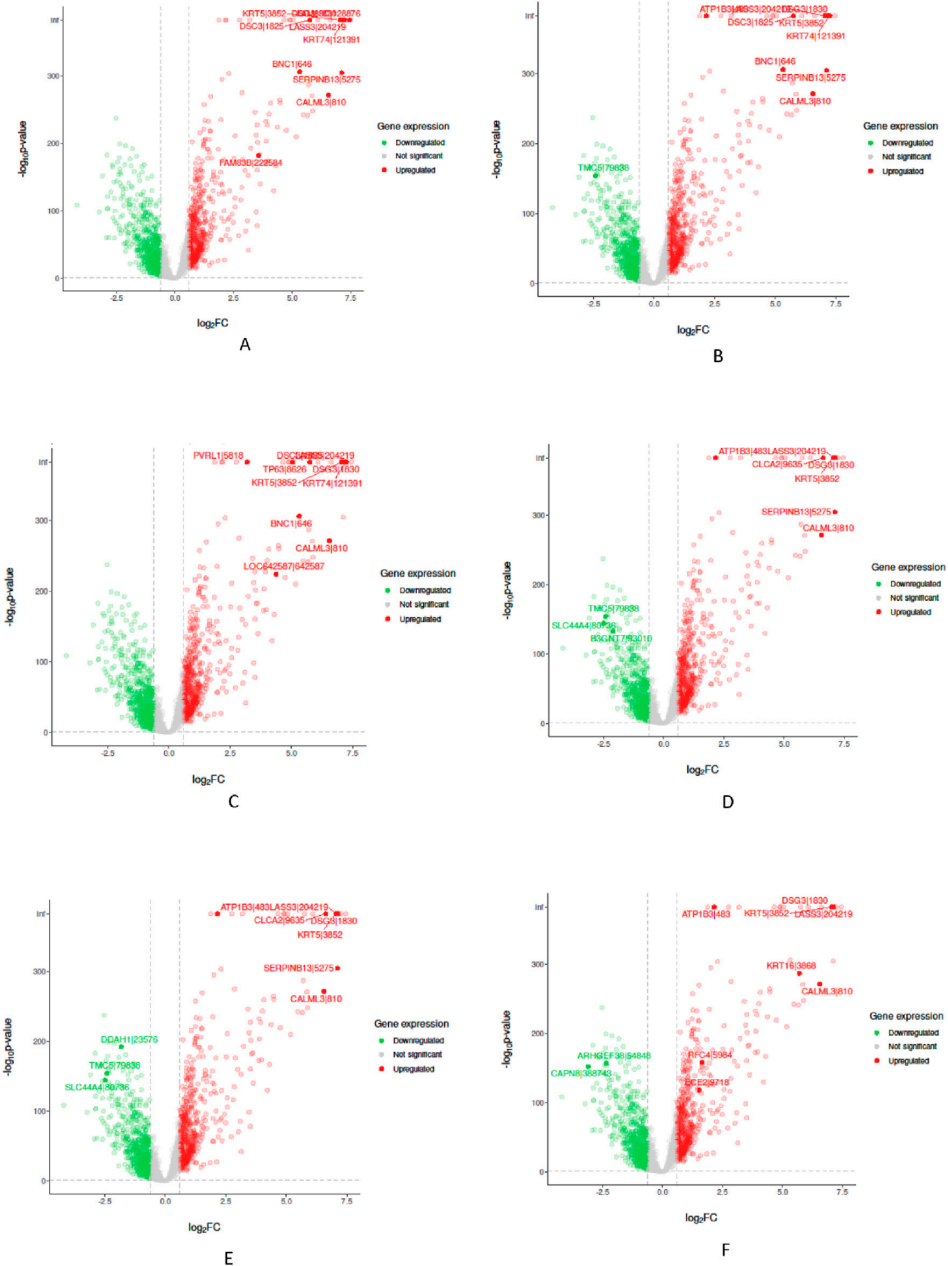
Volcano plots for Random Forest (RF) and eXtreme Gradient Boosting (XGB) models displaying selected feature importance in lung data. Each plot utilizes a different interpretability method, namely LIME, SHAP, and Variable Importance (VarImp), to highlight the most influential features. The *x*-axis shows the log fold change (logFC), indicating the magnitude of change in gene expression, while the *y*-axis represents the negative logarithm of the p-value (−log10 p-value), denoting statistical significance. The plots differentiate between significant (red), non-significant (green), and unregulated (gray) gene expressions. **(A)** RF LIME - Random Forest model feature importance using LIME explanation method. **(B)** RF SHAP - Random Forest model feature importance using SHAP values for explanation. **(C)** RF VarImp - Random Forest model feature importance using Variable Importance. **(D)** XGB LIME - eXtreme Gradient Boosting model feature importance using LIME explanation. **(E)** XGB SHAP - eXtreme Gradient Boosting model feature importance using SHAP values for explanation. **(F)** XGB VarImp - eXtreme Gradient Boosting model feature importance using Variable Importance.

In the “Biological Validation”, we utilize Volcano plots to graphically illustrate the significant gene expressions identified during the XAI analysis. Figures 5, 6 in showcase these pivotal findings for RCC and lung cancer data respectively. The plots incorporate algorithms such as random forest and XGBoost in conjunction with interpretability methods like LIME, SHAP, and Variable Importance. Each plot is methodically constructed with the *x*-axis representing the log fold change (logFC) and the *y*-axis depicting the negative logarithm of the *p*-value (-log10 *p*-value). This arrangement accurately measures the degree of changes in gene expression and their statistical importance. As expected, none of the top genes identified as influential features are located within the gray zone of these plots, which signifies non-significant gene expression. This absence of features in the gray zone underscores the distinct and substantial impact that these genes have in relation to kidney and lung cancer, as determined by the advanced feature selection techniques employed in our study.

**FIGURE 7:**
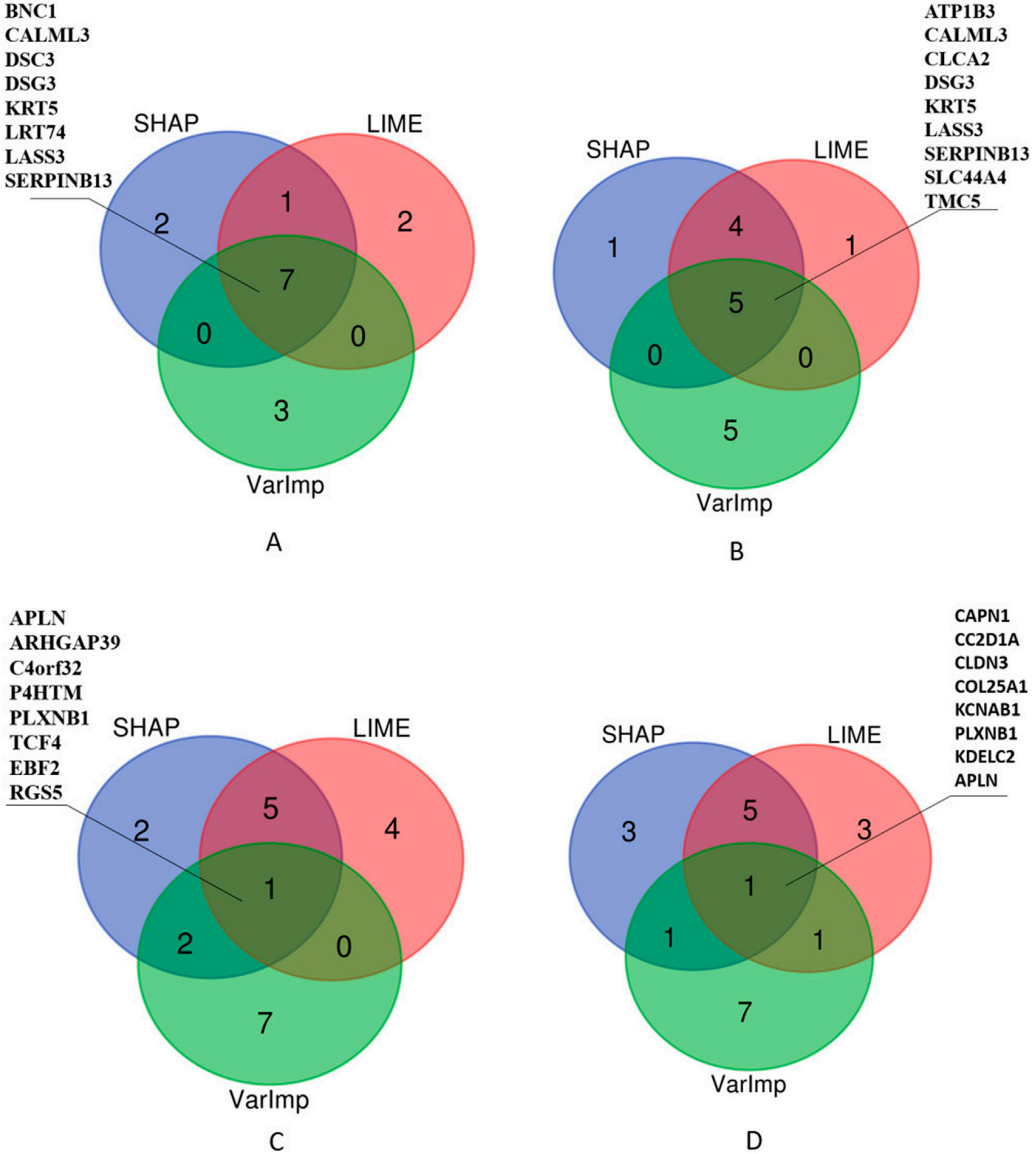
Comparison of XAI methods across different models and datasets. The Venn diagrams illustrate the overlap and uniqueness of selected features as determined by SHAP (SHapley Additive exPlanations), LIME (Local Interpretable Modelagnostic Explanations), and Variable Importance (VarImp) for Random Forest (RF) and eXtreme Gradient Boosting (XGB) models on Lung and RCC datasets. Each number within the diagrams represents the count of features identified by the corresponding methods.

Additionally, we selected the top 10 important features and listed those selected by at least two algorithms in Figure 7. The Venn diagrams presented in Figure 7 offer a visual comparison of the interpretability methods applied to the Random Forest (RF) and XGBoost (XGB) models across Lung and RCC datasets. These diagrams illustrate the features selected by at least two methods, namely, SHAP, LIME, and VarImp. For the RF Lung model (a), we see that eight features were identified by at least two methods as important, suggesting a strong consensus on these features’ influence on the model’s predictions: BNC1, CALML3, DSC3, DSG3, KRT5, LRT74, LASS3, and SERPINB13. In the case of the XGB Lung model (b), nine features were identified by all three methods as important, suggesting a strong consensus on these features’ influence on the model’s predictions: ATP1B3, CALML3, CLCA2, DSG3, KRT5, LASS3, SERPINB13, SLC44A4, and TMC5. For the RF RCC model (c), at least two methods selected APLN, ARHGAP39, C4orf32, P4HTM, PLXNB1, TCF4, EBF2, and RGS5. Lastly, the XGB RCC model (d) shows a notable distribution with eight features, CAPN1, CC2D1A, CLDN3, COL25A1, KCNAB1, PLXNB1, KDELC2, and APLN, identified by VarImp, SHAP, and LIME.

As illustrated in Table 1, given that both methods exhibited performances that were roughly equivalent to each other, we investigated the efficacy of the genes located at the intersection of these two methods, as depicted in Figure 7, by analyzing their effectiveness in accordance with studies in the literature, as will be seen in Table 2.

**TABLE 2:** Differential Gene Expression in Lung and Renal Cell Carcinoma Tissue. The table outlines the regulation patterns of specific genes in lung (Lung XGB, Lung RF) and renal cell carcinoma (RCC XGB, RCC RF) tissues. Gene symbols, their full names, and references for the associated studies are provided alongside the regulation status. Genes are selected by at least two methods: SHAP, LIME, and VarImp.

Analysis	Gene symbol	Real gene name	References	Up/Downregulated
Lung XGB	ATP1B3	ATPase $Na^{+}$ / $K^{+}$ Beta 3	Relli et al. (2018)	Upregulated
	CALML3	Calmodulin Like 3	Zhan et al. (2015)	
	CLCA2	Chloride Channel Accessory 2	Shinmura et al. (2014)	
	DSG3	Desmoglein 3	Zhan et al. (2015)	Upregulated
	KRT5	Keratin 5	Yuan et al. (2020)	Upregulated
	LASS3	Ceramide Synthase 3	Su et al. (2019)	Upregulated
	SERPINB13	Serpin Family B Member 13	Yuan et al. (2020)	Upregulated
	SLC44A4	Solute Carrier Family 44 Member 4	Arroyo et al. (2020)	Downregulated
	TMC5	Transmembrane Channel Like 5	Zhan et al. (2015)	Downregulated
Lung RF	BNC1	Basonuclin 1	Kim et al. (2010), Yuan et al. (2020)	Upregulated
	CALML3	Calmodulin Like 3	Zhan et al. (2015)	Upregulated
	DSC3	Desmocollin 3	Zhan et al. (2015)	Upregulated
	DSG3	Desmoglein 3	Zhan et al. (2015)	Upregulated
	KRT5	Keratin 5	Yuan et al. (2020)	Upregulated
	KRT74	Keratin 74	Keogh et al. (2022)	Upregulated
	LASS3	CERS3	Dwivedi et al. (2023)	Upregulated
	SERPINB13	Serpin Family B Member 13	Yuan et al. (2020)	Upregulated
RCC XGB	CAPN1	Calpain 1		Downregulated
	CC2D1A	Coiled-Coil and C2 Domain Containing 1A		Downregulated
	CLDN3	Claudin 3	Lechpammer et al. (2008)	
	COL25A1	Collagen Type XXV Alpha 1 Chain		Upregulated
	KCNAB1	Potassium Voltage-Gated Channel Subfamily	Sun et al. (2022)	
		A Regulatory Beta Subunit 1		
	PLXNB1	Plexin B1	Gómez-Román et al. (2010), Li et al. (2021)	Downregulated
	KDELC2	KDEL (Lys-Asp-Glu-Leu) Containing 2		Upregulated
	APLN	Apelin	Tolkach et al. (2019)	Upregulated
RCC RF	APLN	Apelin	Tolkach et al. (2019)	Upregulated
	ARHGAP39	Rho GTPase Activating Protein 39	Yao et al. (2023)	Downregulated
	C4orf32	Chromosome 4 Open Reading Frame 32		Upregulated
	P4HTM	Prolyl 4-Hydroxylase, Transmembrane		Downregulated
	PLXNB1	Plexin B1	Gómez-Román et al. (2010), Li et al. (2021)	Downregulated
	TCF4	T cell Factor 4	Zhao et al. (2016), Xu et al. (2016), Lin et al. (2000)	Upregulated
	EBF2	Early B-Cell Factor 2		Upregulated
	RGS5	Regulator of G Protein Signaling 5	Su and Shahriyari (2020)	

In Table 2, we present a summarized overview of gene regulation patterns discernible in lung tissue and RCC, which are listed among the intersected genes in Figure 7. This table is pivotal for delineating the differential expression of genes, as it systematically catalogs those that are upregulated and, notably, the singular gene that is downregulated in the context of RCC. Our analysis employed two distinct methodologies, denoted as XGB and RF, to analyze gene expression patterns across lung and RCC tissues. The ‘Up/Downregulated’ column indicates whether a gene is expressed at higher (upregulated) or lower (downregulated) levels than a predetermined baseline, which in this context is the normal tissue expression level. For lung tissue, the uniform upregulation across both analytical methods (XGB and RF) highlights a consistent over-expression of genes like ATP1B3, which encodes the beta-3 subunit of the $Na^{+}$ / $K^{+}$ -ATPase, and CALML3, which is associated with the calmodulin-like protein family. Similarly, keratin-associated genes KRT5 and KRT74 also exhibit upregulation, suggesting a possible link to structural or regulatory changes in lung tissue under pathological conditions. In contrast, the RCC tissue analysis reveals an intriguing pattern: while the majority of genes such as PLXNB1, associated with cellular structure and signaling, are upregulated, TCF4 stands out as the sole gene that is downregulated. TCF4, or Transcription Factor 4, is a pivotal element of the Wnt signaling pathway, and its downregulation could imply a significant deviation from normal cellular signaling and transcriptional regulation in RCC.

Feature selection in RNA-Seq studies is typically performed using well-established modeling techniques available in R/BIOCONDUCTOR packages like DESeq2 and edgeR. However, these methods are not specifically designed to identify features that contribute most effectively to classification models. Some features, despite being differentially expressed between comparison groups, may not significantly improve the predictive power of a fitted model. To address this, we proposed a hybrid feature selection methodology that aims to select features that are both differentially expressed and contribute meaningfully to the classification model’s performance.

While our methodology is robust, its effectiveness may be influenced by factors such as sample size, feature count, and overdispersion in the data. A comprehensive simulation study, particularly one focusing on Type-I and Type-II error rates, would be valuable for further evaluating these factors. However, this falls beyond the current scope of our study and could be explored in future research. The limitations mentioned have been acknowledged within the manuscript.

## 4 Conclusion

This study introduced a ML-GAP that utilizes autoencoders and data augmentation to enhance the detection and interpretation of DEGs from RNA-Seq count data. The overarching goal was to address the limitations of traditional RNA-Seq analysis methods by incorporating advanced ML techniques, thereby providing a deeper understanding of gene expression patterns and their associations with clinical outcomes. The ML-GAP workflow includes several key steps: data preprocessing to mitigate low-quality features and inter-sample variations; dimensionality reduction through PCA and DEGs identification; and data augmentation to increase the robustness and generalizability of the ML models. Notably, the MixUp augmentation method demonstrated superior results, especially in the context of RCC, indicating its effectiveness in enhancing the predictive accuracy of our models. Therefore, we further analyzed the results obtained from the MixUp method in the next step XAI. The application of XAI techniques, such as SHAP and LIME, has been crucial in ensuring the transparency and interpretability of our findings. These methods allowed us to identify the most impactful features, aligning with our objective to not only predict but also understand the biological significance behind the predictions. The integration of GO analysis further validated the relevance of these features in the context of lung cancer and RCC, underscoring the biological and clinical implications of our results.

The ML-GAP represents a significant advance in genomic analysis, combining the strengths of ML, data augmentation, and interpretability to uncover novel insights into gene expression dynamics. However, challenges such as data quality, computational demands, and the need for further methodological refinement remain. Future work will focus on addressing these challenges, exploring additional datasets, and continuing to enhance the pipeline’s accuracy and applicability to various genomic contexts. Additionally, in future work, it will be valuable to explore the use of generative deep learning models for generating realistic bulk RNA-Seq gene expression data. Similar to Wang et al.‘s study (Wang et al., 2024), which used Generative Adversarial Networks (GANs) to produce augmented datasets and analyzed them with SHAP, integrating these models into our ML-GAP pipeline could significantly enhance data augmentation and improve downstream analyses in high-throughput transcriptomics.

## Data Availability

Publicly available datasets were analyzed in this study. This data can be found here: https://www.cancer.gov/ccg/research/genome-sequencing/tcga.

## Abbreviations

DEGs, Differentially Expressed Genes; LIME , Local Interpretable Model-agnostic Explanations; LUAD, Lung Adenocarcinoma; LUSC, Lung Squamous Cell Carcinoma; ML, Machine Learning; ML-GAP, Machine Learning-Enhanced Genomic Data Analysis Pipeline; PCA, Principal Component Analysis; RCC, Renal Cell Carcinoma; RNA-Seq, RNA Sequencing; SHAP, SHapley Additive exPlanations; TCGA, The Cancer Genome Atlas; VarImp, Variable Importance; XAI, explainable artificial intelligence.
