# Supplementary material for: ML-GAP: machine learning-enhanced genomic analysis pipeline using autoencoders and data augmentation
Source: Front Genet. 2024 Sep 25;15:1442759. doi: 10.3389/fgene.2024.1442759 (PMC11467662; doi:10.3389/fgene.2024.1442759)
Supplement: Supplementary file 1 [file DataSheet1.PDF]

frontiers

1

ML-GAP: Machine Learning-Enhanced Genomic Analysis Pipeline with Autoencoders and Data Augmentation

Melih Agraz [1,2*], **Dincer Goksuluk** [3], **Peng Zhang** [4,5], **Bum-Rak Cho** [5,7], **Richard T. Clements** [6], **Gaurav Choudhary** [4,5,7], **George Em Karniadakis** [1,8],

[1] *Division of Applied Mathematics, Brown University, Providence, 02906, RI, US*
[2] *Department of Statistics, Giresun University, Giresun, 28200,Turkiye*
[3] *Department of Biostatistics, Erciyes University, Melikgazi/Kayseri, 38039, Türkiye*
[4] *Vascular Research Laboratory, VA Providence Healthcare System, Providence, 02903, RI, US*
[5] *Division of Cardiology, Department of Medicine, Alpert Medical School of Brown University, Providence, 02903, RI, US*
[6] *Department of Biomedical and Pharmaceutical Sciences, University of Rhode Island College of Pharmacy, South Kingston, 02881, RI, US*
[7] *Cardiovascular Research Center, Rhode Island Hospital, Rhode Island, 02903, RI, US*
[8] *School of Engineering, Brown University, Rhode Island, 02912, RI, US*

Correspondence*:
Corresponding Author
melih_agraz@brown.edu

Abbreviation	Full Form
SHAP	SHapley Additive exPlanations
TCGA	The Cancer Genome Atlas
LUAD	Lung Adenocarcinoma
LUSC	Lung Squamous Cell Carcinoma
LIME	Local Interpretable Model-agnostic Explanations
VarImp	Variable Importance
RCC	Renal Cell Carcinoma
PCA	Principal Component Analysis
RNA-Seq	RNA Sequencing
DEGs	Differentially Expressed Genes
ML	Machine LEarning
XAI	explainable artificial intelligence
ML-GAP	Machine Learning-Enhanced Genomic Data Analysis Pipeline

Table 1. List of Abbreviations
